# Crystal structure and Hirshfeld surface analysis of {2-[bis­(pyridin-2-ylmeth­yl)amino]­ethane-1-thiol­ato}­chlorido­cadmium(II)

**DOI:** 10.1107/S2056989024009198

**Published:** 2024-09-30

**Authors:** Todd M. Reynolds, Steven M. Berry, Deborah C. Bebout

**Affiliations:** a Department of Chemistry, William & Mary, Williamsburg, VA 23187-8795, USA; Universität Greifswald, Germany

**Keywords:** crystal structure, Hirshfeld surface analysis, chelating *N*,*S*-ligands, Cd^2+^ complex

## Abstract

The title compound, [Cd(C_14_H_16_N_3_S)Cl] or [Cd**L**Cl] (**1**), where **LH** = 2-[bis­(pyridin-2-ylmeth­yl)amino]­ethane-1-thiol, was prepared and structurally characterized. The Cd^2+^ complex crystallizes in *P*2_1_/*c* with a distorted trigonal–bipyramidal metal coordination geometry. Supra­molecular inter­actions in **1** include parallel offset face-to-face inter­actions between inversion-related pyridyl rings and potential hydrogen bonds with chlorine or sulfur as the acceptor. Additional cooperative pyrid­yl–pyridyl inter­actions with roughly 45° tilt angles and centroid–centroid distances of less than 5.5 Å likely also contribute to the overall solid-state stability. Hirshfeld surface analysis indicates that H⋯H (51.2%), Cl⋯H/H⋯Cl (13.9%), C⋯H/H⋯C (12.3%) and S⋯H/H⋯S (11.8%) inter­actions are dominant in the solid state.

## Abstract 2

A mononuclear cadmium(II) complex [Cd**L**Cl], where **HL** = 2-[bis­(pyridin-2-ylmeth­yl)amino]­ethane-1-thiol, was synthesized and characterized by single-crystal X-ray diffraction and Hirshfeld analysis.

## 1. Chemical context

The cambialistic ζ-class of carbonic anhydrases from marine diatoms relying on Cd^2+^ as their metal cofactor when Zn^2+^ is scarce were discovered in 2000 (Lane & Morel, 2000 ▸). These proteins have His_2_Cys metal-binding environments like the prokaryotic β-class of Zn^2+^-dependent carbonic anhydrases (Xu *et al.*, 2008 ▸). Despite the concurrence of histidine and cysteine in the active site of these proteins associated with the only known physiologically beneficial role for Cd^2+^, structurally characterized complexes of Cd^2+^ with chelating ligands containing a combination of aromatic amine and alkyl­thiol­ate donors remain rare (CSD, Version 5.45, update of June 2024; Groom *et al.*, 2016 ▸) and include only multinuclear complexes (Sturner *et al.*, 2024 ▸; Brennan *et al.* 2022 ▸; Lai *et al.* 2013 ▸). Herein, the preparation, crystal structure and Hirshfeld surface analysis of mononuclear {2-[bis­(pyridin-2-ylmeth­yl)amino]­ethane-1-thiol­ato}chlorido­cadmium(II) are reported.
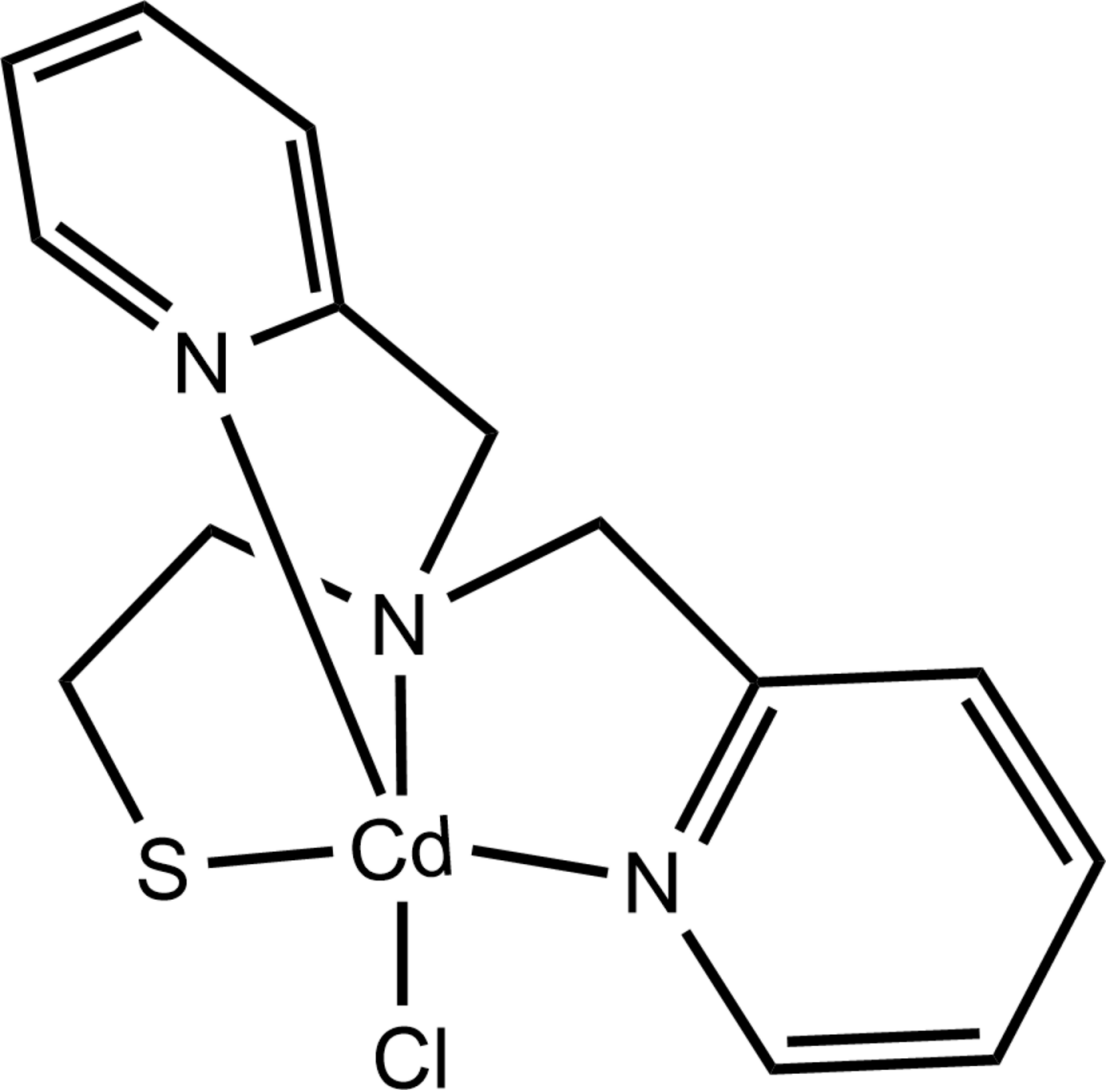


## 2. Structural commentary

**Figure 1:**
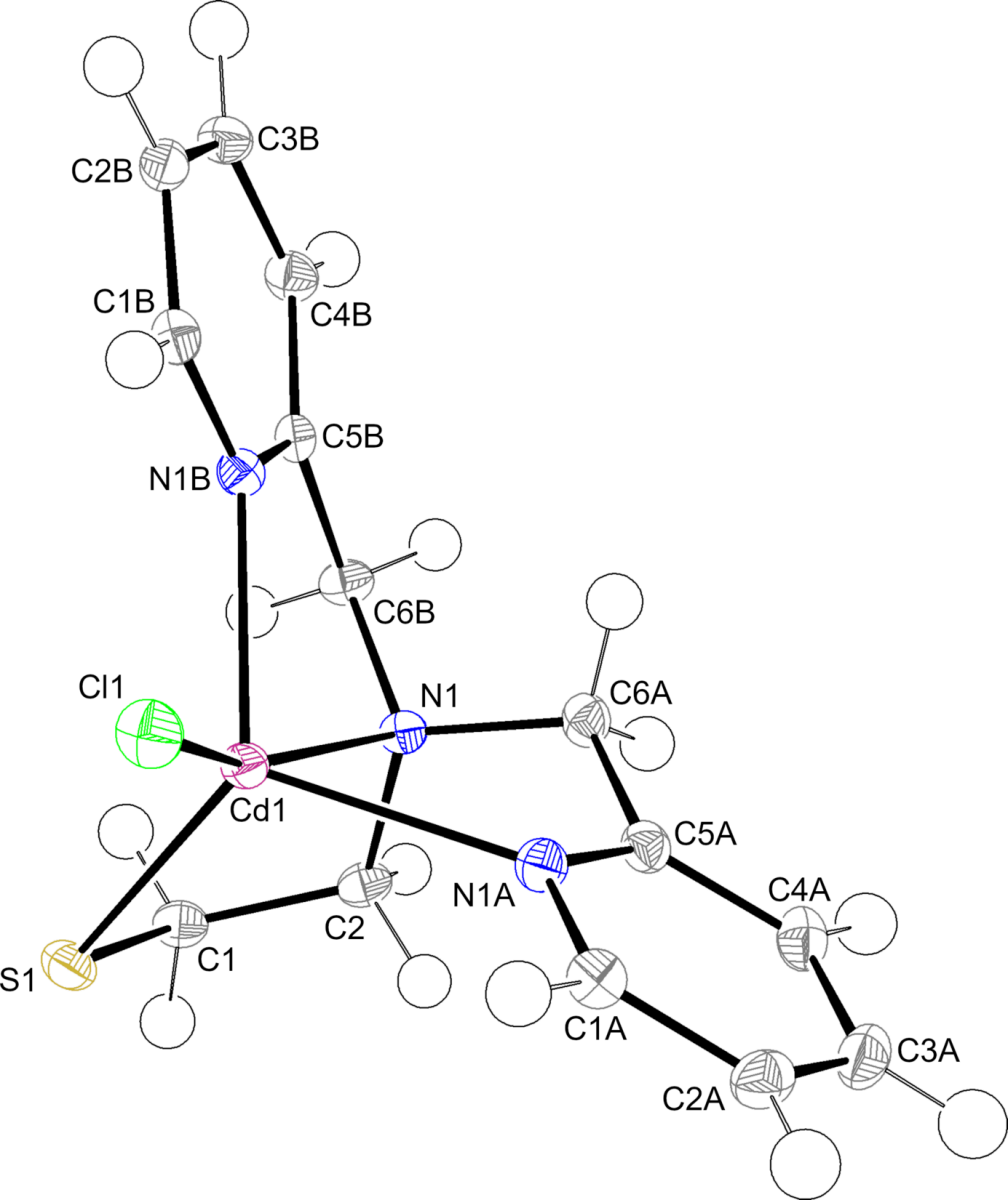
The mol­ecular structure of **1** with the atom-numbering scheme generated with *ORTEP-3 for Windows* (Farrugia, 2012 ▸). Displacement ellipsoids are drawn at the 50% probability level.

Complex **1** crystallizes from methanol/*n*-butanol with NaOH as a base in the monoclinic space group *P*2_1_/*n* as a monomer (Fig. 1 ▸) instead of the dimer previously isolated from either methanol/benzene or methanol/ethyl acetate (Sturner *et al.*, 2024 ▸). The asymmetrically coordinated tetra­dentate organic ligand and one chloride provide a predominantly trigonal–bipyramidal coordination geometry (τ = 0.75; Table 1 ▸) to the metal ion (Addison *et al.*, 1984 ▸). The N1 and Cl1 atoms define the axial positions with a N1—Cd1—Cl1 bond angle of 161.29 (3)°. The cadmium atom is 0.6107 (6) Å above the mean N1*A*–N1*B*–S1 trigonal plane, away from the axial N1 atom, and closer to the axial Cl1 atom. The three chelate rings have envelope conformations with either N1, C6*A* or C1 in the flap positions.

## 3. Supra­molecular features

**Figure 2:**
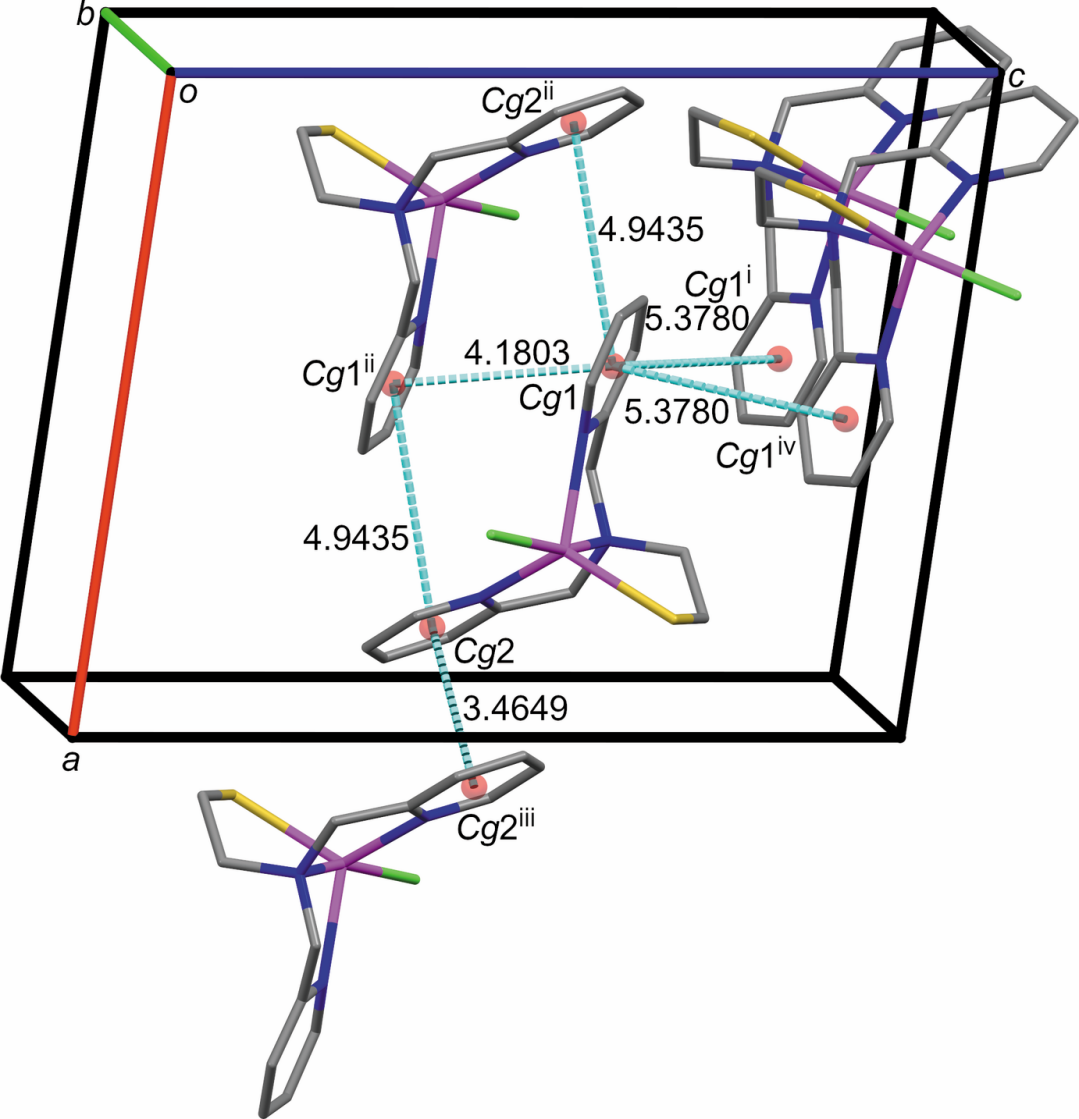
Centroid–centroid distances between nearby pyridyls in **1** viewed down the *b* axis illustrated using *Mercury* (Macrae *et al.*, 2020 ▸). Hydrogen atoms are omitted for clarity. Ring centroids are shown as red spheres. For additional numerical data, see Table 2 ▸. Symmetry codes: (i) 1 − *x*, 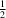 + *y*, 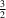 − *z*; (ii) 1 − *x*, 1 − *y*, 1 − *z*; (iii) 1 − *x*, −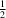 + *y*, 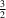 − *z*; (iv) 2 − *x*, 1 − *y*, 1 − *z*.

**Figure 3:**
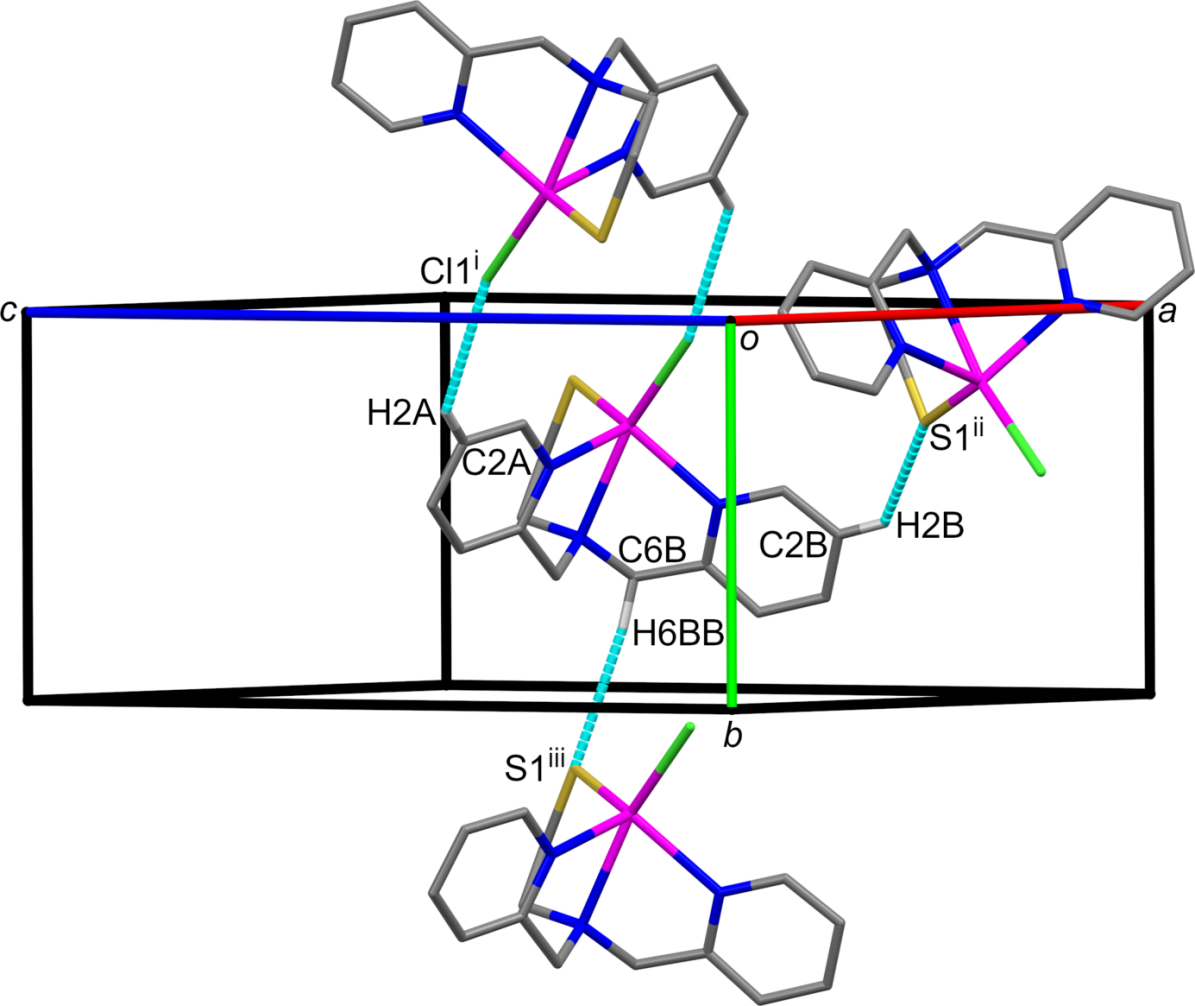
A view of the C—H⋯Cl and C—H⋯S hydrogen bonds in compound **1**, shown as cyan dashed lines illustrated using *Mercury* (Macrae *et al.*, 2020 ▸). Only hydrogen atoms involved in hydrogen bonds are shown for clarity. Symmetry codes as in Table 3 ▸.

**Figure 4:**
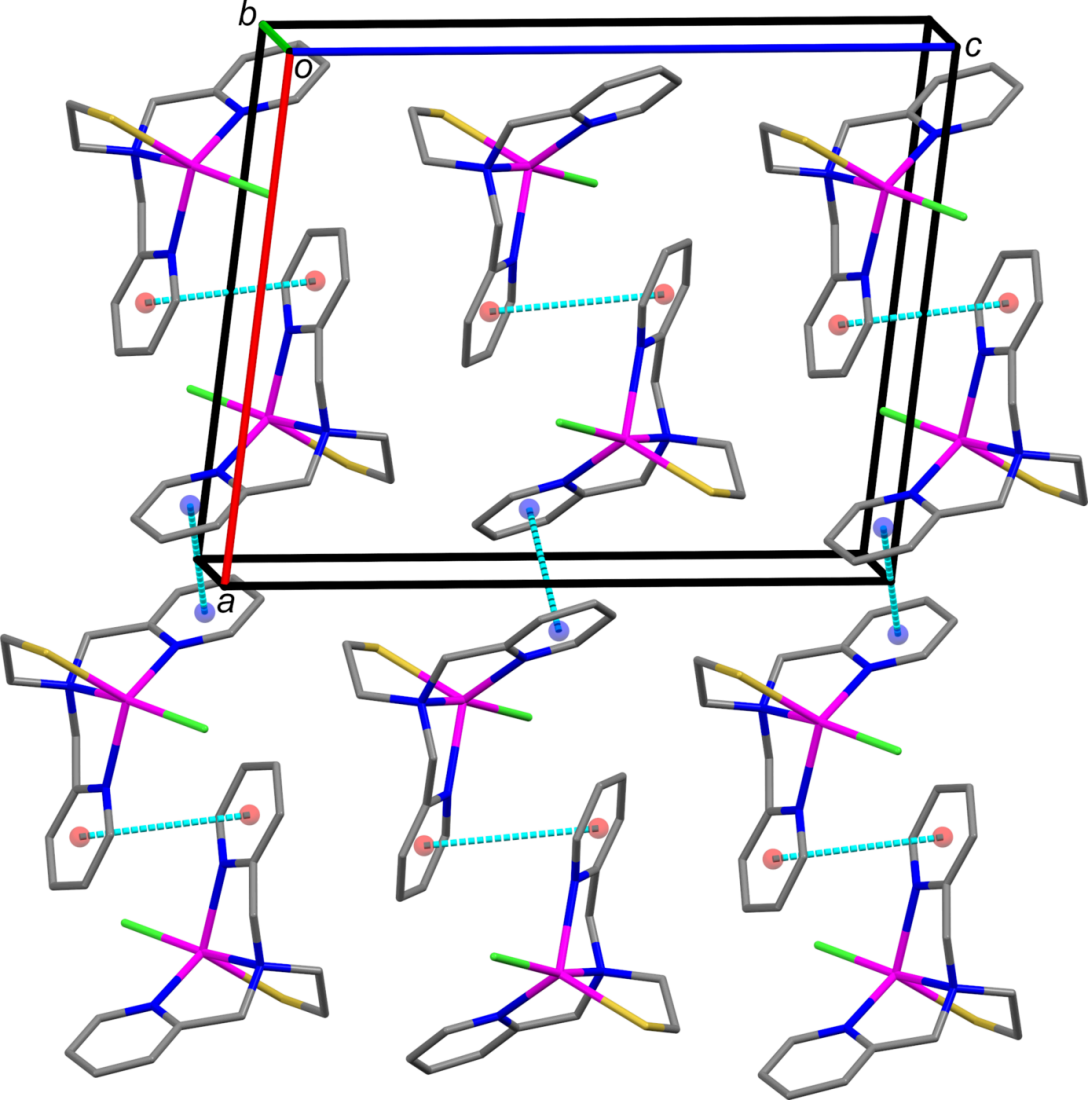
Offset parallel face-to-face π–π stacking inter­actions in **1** between the following ring centroids (*Cg*) shown as colored spheres: *Cg*1 (N1*A*/C1*A*–C5*A*, red sphere); *Cg*2 (N1*B*/C1*B*–C5*B*, blue sphere).

The packing of **1** is stabilized by π–π stacking inter­actions (Fig. 2 ▸; Table 2 ▸), hydrogen bonding (Fig. 3 ▸; Table 3 ▸) and van der Waals inter­actions (Table 4 ▸). One face of each ligand pyridyl ring is stacked against an inversion-related equivalent with a small offset (Table 2 ▸), creating one-dimensional strands of mol­ecules along the *a-*axis direction (Fig. 4 ▸). Inter­estingly, the N1*A* pyridyl rings have roughly 45° tilt angles and centroid–centroid distances of less than 5.5 Å with both the N1*B* pyridyl rings within the strands and N1*A* pyridyl rings of adjacent strands. Stabilizing contributions from these hybrid offset face-to-face/edge-to-face inter­actions are supported by a quantum chemistry study of the benzene dimer associating a tilt angle of about 45° with a shallow minimum on the path inter­converting offset-parallel benzene dimers through a perpendicular saddle point (Jaffe & Smith, 1996 ▸). Furthermore, structural analysis of aromatic ligands bound to proteins found an abundance of phenyl­alanine and tyrosine residues with comparable ring orientation metrics (Brylinski, 2018 ▸).

Both metal-bound chlorine (Aullón *et al.*, 1998 ▸) and sulfur atoms (Chand *et al.*, 2020 ▸) serve as hydrogen-bond acceptors in **1**. Pairs of inversion-related mol­ecules connected by C—H⋯Cl hydrogen bonds are stacked along the *b* axis. The C—H⋯S hydrogen bonds form sheets of mol­ecules in the *bc* plane.

## 4. Hirshfeld surface analysis

**Figure 5:**
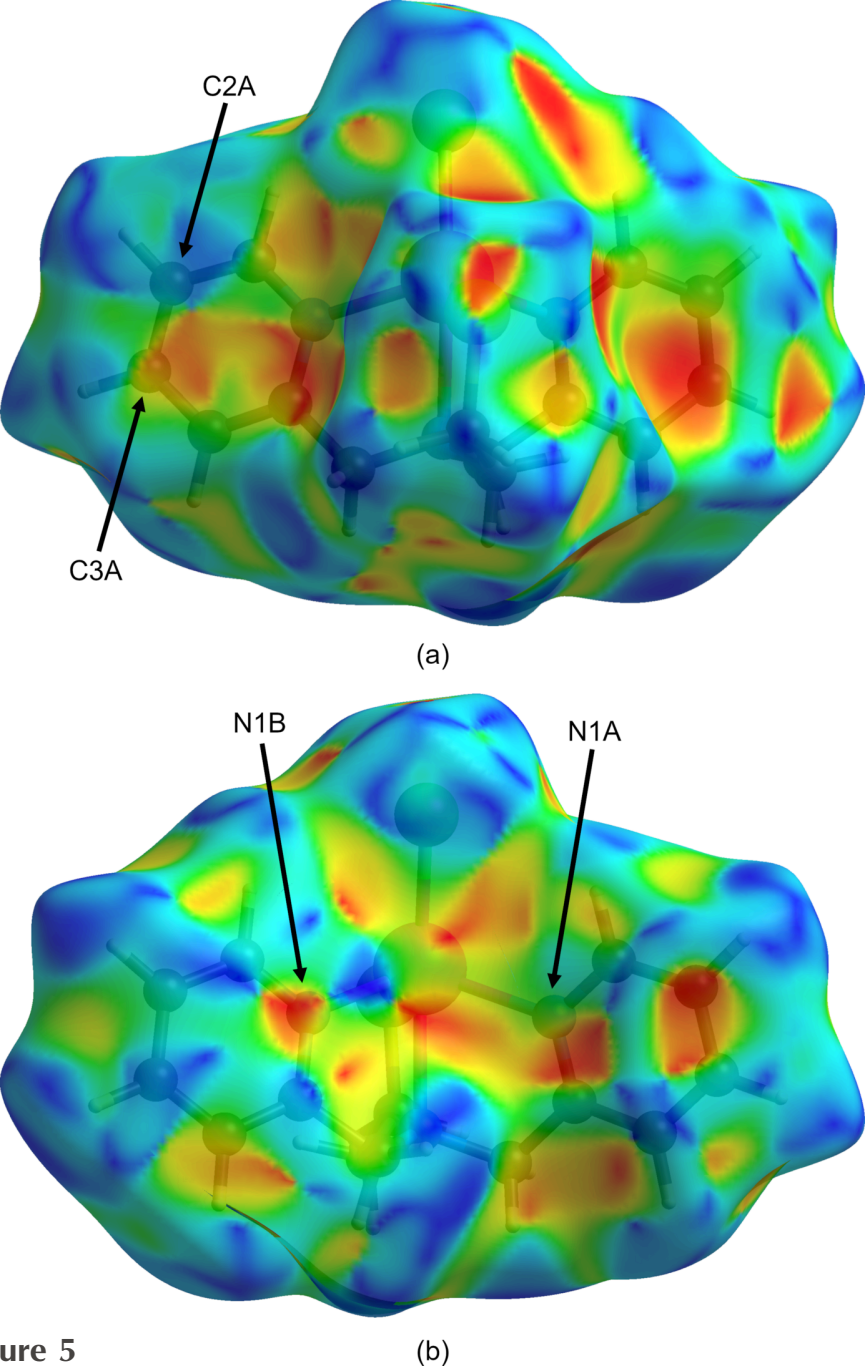
Hirshfeld surface of **1** plotted over the shape-index for two orientations generated with *Crystal Explorer 21.5* (Spackman *et al.*, 2021 ▸) with the ethyl­thiol­ato group to the (*a*) front and (*b*) back. Red and blue areas represent hollow and bump regions, respectively, on the shape-index surface.

Inter­molecular inter­actions were investigated by qu­anti­tative analysis of the Hirshfeld surface and visualized with *Crystal-Explorer 21.5* (Spackman *et al.*, 2021 ▸). The Hirshfeld surface of **1** plotted over the shape-index has hourglass figures associated with parallel face-to-face aromatic inter­actions over the C2*A*–C3*A* edge of the N1*A* pyridyl ring (Fig. 5 ▸*a*) and N1*B* atom (Fig. 5 ▸*b*). A pair of arc-shaped blue bumps associated with the periphery of the N1*B* pyridyl ring have complementary inversion-related red hollows surrounding the Cd—N1*A* bond (Fig. 5 ▸*b*). The blue-streaked dome associated with the chlorine atom nestles against the red hollow below the N1*B*—C5*B* bond (Fig. 5 ▸*b*).

**Figure 6:**
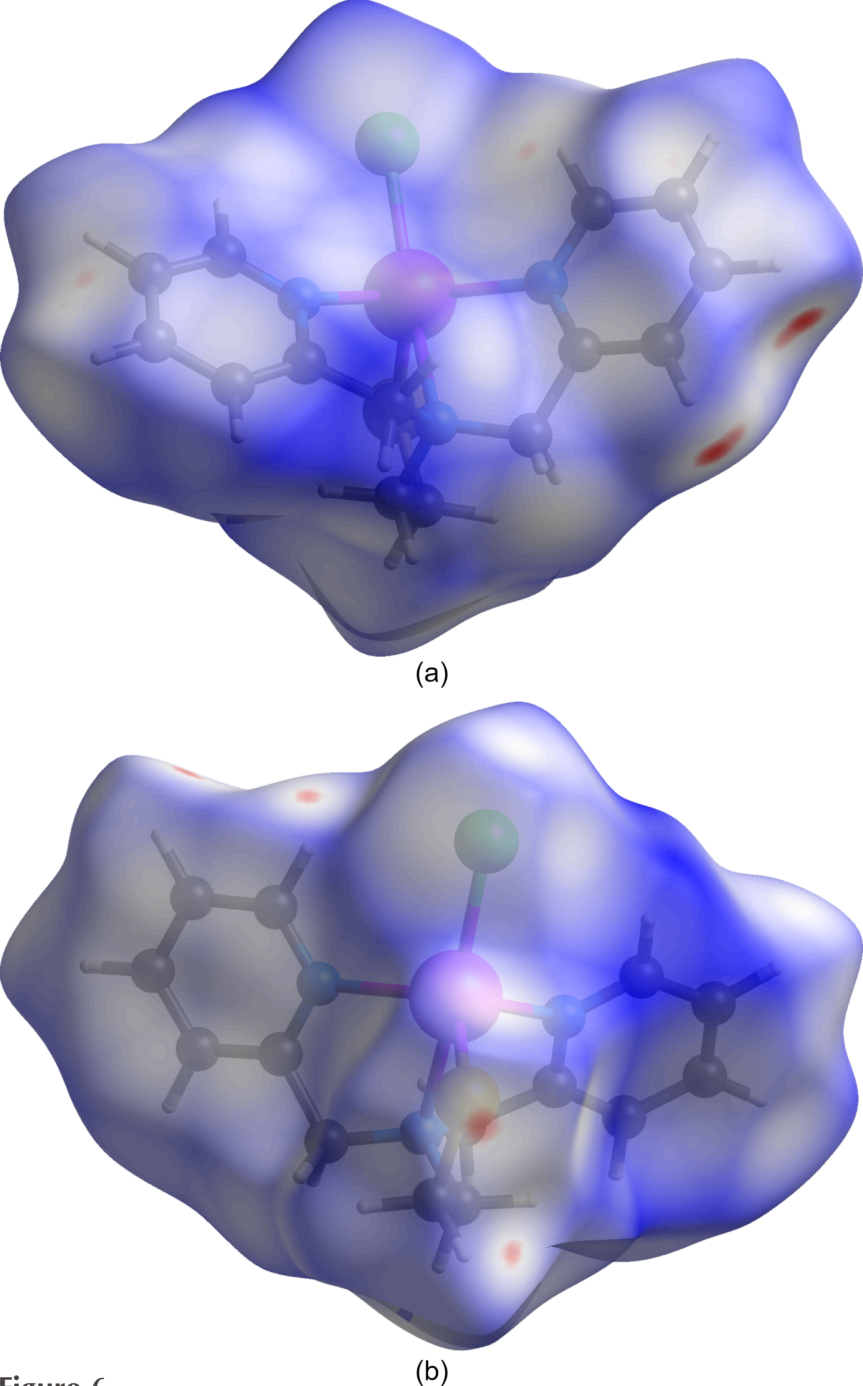
Views of the Hirshfeld surface of **1** plotted over normalized contact distance (*d*_norm_) with the ethyl­thiol­ato group to the (*a*) back and (*b*) front. The plot was generated using *Crystal Explorer 21.5* (Spackman *et al.*, 2021 ▸) with *d*_norm_.

The Hirshfeld surface of **1** mapped with the function *d*_norm_, the sum of the distances from a surface point to the nearest inter­ior (*d*_i_) and exterior (*d*_e_) atoms normalized by the van der Waals (vdW) radii of the corresponding atom (rvdW), is shown in Fig. 6 ▸. Contacts shorter than the sums of vdW radii are shown in red, those longer in blue, and those approximately equal as white areas. The most intense red spots correspond to close contacts between C3*B*, H3*B* and H4*B* along the pyridyl edges of inversion-related mol­ecules (Fig. 6 ▸*a*). Atoms H1*B* and H2*B* of the same pyridyl ring form close contacts with H1*CA* and S1 of a single neighboring mol­ecule (Fig. 6 ▸*b*). Additional faint spots associated with a close contact between C1*B* and both C3*A* and H3*A* are also observed (Fig. 6 ▸*a*). The remaining close contacts cause very faint red spots.

**Figure 7:**
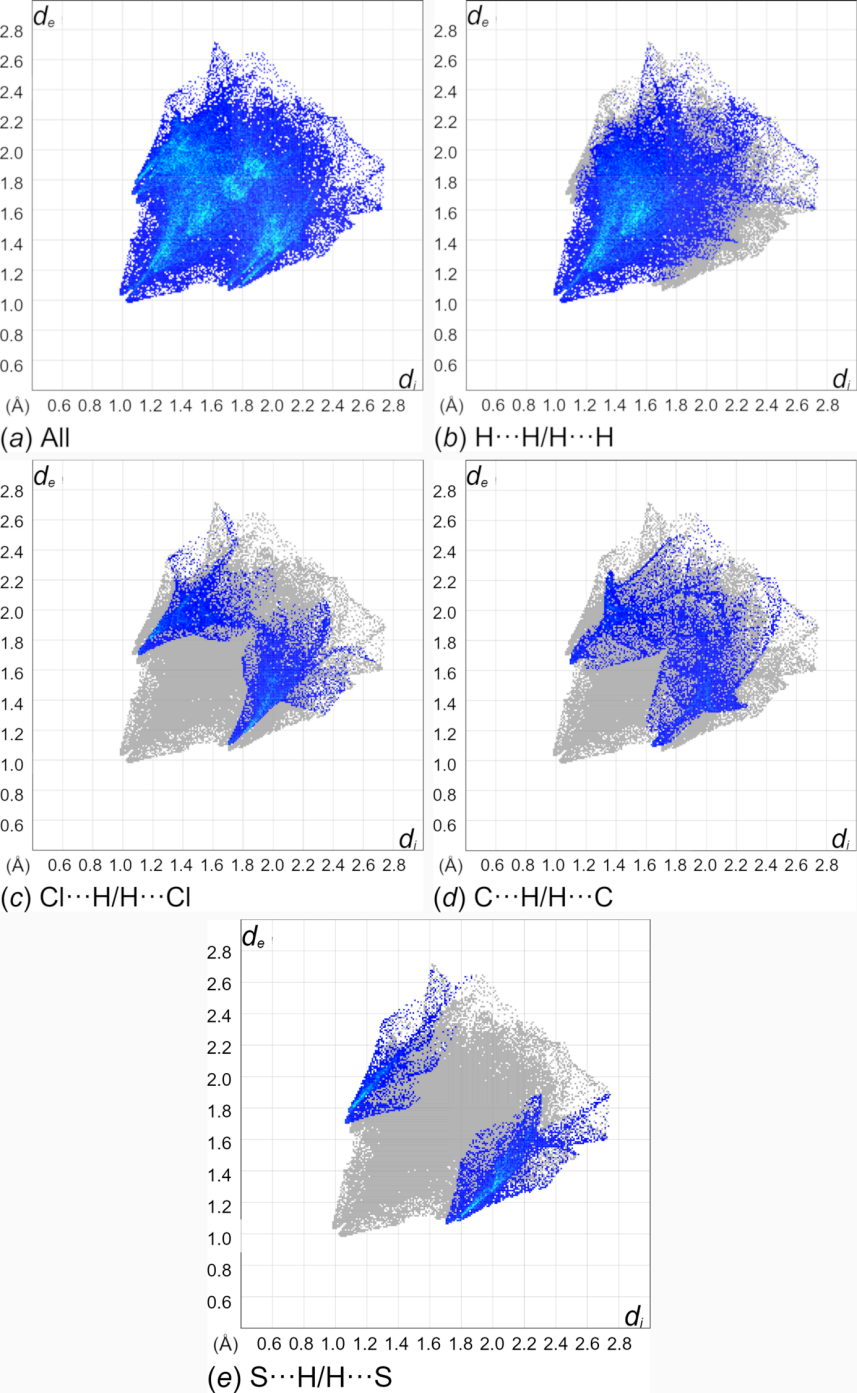
The full two-dimensional fingerprint plots for **1**, showing (*a*) all inter­actions, and components delineated into (*b*) H⋯H, (*c*) Cl⋯H/H⋯Cl, (*d*) C⋯H/H⋯C and (*e*) S⋯H/H⋯S inter­actions generated with *Crystal Explorer 21.5* (Spackman *et al.*, 2021 ▸). The *d*_i_ and *d*_e_ values are closest inter­nal and external distances (in Å) from given points on the Hirshfeld surface.

The overall 2D fingerprint plot for **1** is provided in Fig. 7 ▸*a.* Breakdown by element indicated H⋯H (51.2%) are predominant, followed by comparable amounts of Cl⋯H/H⋯Cl (13.9%), C⋯H/H⋯C (12.3%) and S⋯H/H⋯S (11.8%) inter­actions (Fig. 6 ▸*b*–*e*). Other minor contributions to the Hirshfeld surface are from N⋯C/C⋯N (3.8%), C⋯C (2.1%), Cl⋯C/C⋯Cl (2.0%), N⋯H/H⋯N (1.8%), Cd⋯H/H⋯Cd (0.7%), N⋯N (0.3%), and Cd⋯C/C⋯Cd (0.1%) contacts.

## 5. Database survey

A search of the Cambridge Structural Database (CSD, Version 5.45, update of June 2024; Groom *et al.*, 2016 ▸) for complexes of cadmium bound to a thiol­ate sulfur, three nitro­gen and one chlorine atoms yielded ten hits, all of which included μ_2_-Cl bridges between cadmium atoms. Three of the complexes are solvomorphs of the μ_2_-Cl_2_ bridged dimer [Cd**L**Cl]_2_ (refcodes BOJTIH, BOJTUT, BOJVAB: Sturner *et al.*, 2024 ▸). Other μ_2_-Cl_2_ bridged dimers included bis­(μ_2_-chlorido)­bis­(2,2′-bi­pyridine-*N*,*N*′)bis­(4,6-di­methyl­pyrimidine-2-thiol­ato-*N*,*S*)dicadmium(II) and bis­(μ_2_-chlorido)­bis­(4,6-di­methyl­pyrimidine-2-thiol­ato-*N*,*S)*bis­(1,10-phenanthroline-*N*,*N′*)dicadmium(II) with N_3_SCl_2_ metal coordination environments (refcodes LUMZOJ and LUMZUP, respectively: Lang *et al.*, 2009 ▸). Four of the structurally characterized complexes were μ_2_-Cl bridged complexes of *N*-alkyl­ated hexa­aza­dithio­pheno­late dinucleating macrocycles (refcode FIMKOC: Lozan & Kersting, 2005 ▸; refcodes KEVXIT, KEVXOZ and KEVXUF: Gressenbuch & Kersting, 2007 ▸). The final complex was the 1D polymer {[Cd_3_(deatrz)_4_Cl_2_(SCN)_4_]·2H_2_O}_*n*_ (deatrz = 3,5-diethyl-4-amino-1,2,4-triazole) constructed of trinuclear cadmium units bridged by both triazole ligands and chloride (refcode EQUHAZ: Yi *et al.*, 2004 ▸).

A further search of the CSD for complexes of Cd^2+^ bound to chelating ligands containing both an aromatic amine and an alkyl­thiol­ate yielded one binuclear complex (Sturner *et al.*, 2024 ▸) and multinuclear complexes [Cd**L**]_3_(ClO_4_)_3_ (refcode BERXUV: Brennan *et al.*, 2022 ▸) and bis­(μ_3_-carbonato)hexa­kis­{μ_2_-*N*-(2-pyridyl­meth­yl)-*N*-[2-(methyl­thio)­eth­yl]-*N*-(2-mer­captoeth­yl)amine}­hexa­cadmium(II) diperchlorate monohydrate (refcode DEZCUI: Lai *et al.*, 2013 ▸). Additional reported Cd^2+^ complexes containing separate aromatic amine and alkyl thiol­ate ligands included bis­(3,5-di­methyl­pyridine)­bis­(tri­phenyl­methane­thiol­ato)cadmium(II) (refcode HABQEJ: Rheingold & Hampden-Smith, 2015 ▸), *catena*-[bis­(μ_2_-5,10,15,20-tetra­kis­(4-pyrid­yl)porphyrinato)bis­(μ_2_-2-mercapto­ethanol)dicadmium(II) di­methyl­formamide solvate] (refcode JITFEY: Zheng *et al.*, 2007 ▸), bis­(μ_2_-oxo-2-eth­oxy­ethane­thiol­ato)bis­(2,2′-bi­pyridine)­diiodidodicadmium(II) (ref­code OJEPOK: Clegg & Fraser, 2016 ▸) and bis­(μ_2_-oxo-2-eth­oxy­ethane­thiol­ato)bis­(2,2′-bi­pyridine)­dibromido­dicad­mium(II) (refcode OJEPUQ: Clegg & Fraser, 2016 ▸).

## 6. Synthesis and crystallization

Literature procedures were used to prepare **LH** (Lai *et al.*, 2013 ▸). One equivalent of 50 m*M* of CdCl_2_ in methanol was added dropwise with stirring to a 50 m*M* solution of **LH** in methanol containing one equivalent of NaOH. *n*-Butanol was added as a cosolvent. After four weeks of slow evaporation, colorless X-ray quality blocks of **1** were obtained.

## 7. Refinement

Crystal data, data collection and structure refinement details are summarized in Table 5 ▸. The hydrogen atoms were placed in calculated positions with C—H distances of 0.95 Å (aromatic) and 0.99 Å (methyl­ene) and refined as riding atoms with *U*_iso_(H) = 1.2*U*_eq_(C).

## Supplementary Material

Crystal structure: contains datablock(s) I. DOI: 10.1107/S2056989024009198/yz2058sup1.cif

Structure factors: contains datablock(s) I. DOI: 10.1107/S2056989024009198/yz2058Isup2.hkl

Supporting information file. DOI: 10.1107/S2056989024009198/yz2058Isup3.cdx

CCDC reference: 2385270

Additional supporting information:  crystallographic information; 3D view; checkCIF report

## Figures and Tables

**Table 1:** Selected geometric parameters (Å, °) for **1**

Cd1—N1*A*	2.3313 (11)	Cd1—S1	2.4710 (4)
Cd1—N1*B*	2.3151 (11)	Cd1—Cl1	2.4674 (4)
Cd1—N1	2.4758 (11)		
			
N1*A*—Cd1—N1*B*	110.87 (4)	N1*B*—Cd1—S1	116.16 (3)
N1*A*—Cd1—N1	71.91 (4)	N1*B*—Cd1—Cl1	100.00 (3)
N1*A*—Cd1—S1	113.78 (3)	S1—Cd1—N1	82.51 (3)
N1*A*—Cd1—Cl1	97.67 (3)	Cl1—Cd1—N1	161.29 (3)
N1*B*—Cd1—N1	70.70 (4)	Cl1—Cd1—S1	116.159 (13)

**Table 2:** Overview of pyrid­yl–pyridyl ring geometry inter­action metrics (Å, °) for **1** *Cg*1 and *Cg*2 are the centroids of the N1*A*/C1*A*–C5*A* and N1*B*/C1*B*–C5*B* rings, respectively.

Centroids	Dihedral angle between rings	Centroid–centroid distance	Centroid–plane distance	Slippage
*Cg*1⋯*Cg*1^i^	48.66 (7)	5.3780 (6)	1.9060 (6)	–
*Cg*1⋯*Cg*1^ii^	0	4.1803 (5)	3.6544 (6)	2.030
*Cg*2⋯*Cg*1^ii^	45.41 (4)	4.9435 (5)	2.0400 (5)	–
*Cg*2⋯*Cg*2^iii^	0	3.4649 (4)	3.3629 (5)	0.834

Symmetry codes: (i) 1 − *x*, 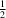 + *y*, 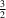 − *z*; (ii) 1 − *x*, 1 − *y*, 1 − *z*; (iii) 2 − *x*, 1 − *y*, 1 − *z*.

**Table 3:** Hydrogen-bond geometry (Å, °) for **1**

*D*—H⋯*A*	*D*—H	H⋯*A*	*D*⋯*A*	*D*—H⋯*A*
C2*A*—H2*A*⋯Cl1^i^	0.95	2.93	3.6784 (15)	137
C2*B*—H2*B*⋯S1^ii^	0.95	2.88	3.5459 (14)	128
C6*B*—H6*BB*⋯S1^iii^	0.99	2.97	3.9133 (13)	160

Symmetry codes: (i) 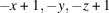; (ii) 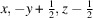; (iii) 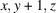.

**Table 4:** Short inter­molecular contacts (Å) for **1**

S1⋯H6*BB*^i^	2.967	H4*B*⋯H3*B*^iv^	2.230
Cl1⋯H2*A*^ii^	2.928	H4*B*⋯H4*B*^iv^	2.395
C1*B*⋯C3*A*^iii^	3.371	H1*B*⋯H1*CA*^v^	2.304
C1*B*⋯H3*A*^iii^	2.820	H2*B*⋯S1^v^	2.877
H4*B*⋯C3*B*^iv^	2.851	C2*B*⋯H2*CB*^vi^	2.847

Symmetry codes: (i) *x*, −1 + *y*, *z*; (ii) 1 − *x*, −*y*, 1 − *z*; (iii) 1 − *x*, 1 − *y*, 1 − *z*; (iv) 2 − *x*, 2 − *y*, 1 − *z*; (v) *x*, 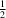 − *y*, −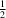 + *z*; (vi) *x*, 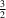 − *y*, −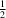 + *z*.

**Table 5:** Experimental details

Crystal data	
Chemical formula	[Cd(C_14_H_16_N_3_S)Cl]
*M*_r_	406.21
Crystal system, space group	Monoclinic, *P*2_1_/*c*
Temperature (K)	100
*a*, *b*, *c* (Å)	12.8451 (14), 7.4508 (7), 15.9284 (17)
β (°)	96.965 (3)
*V* (Å^3^)	1513.2 (3)
*Z*	4
Radiation type	Mo *K*α
μ (mm^−1^)	1.75
Crystal size (mm)	0.37 × 0.31 × 0.24

Data collection	
Diffractometer	Bruker D8 Venture Photon 3
Absorption correction	Multi-scan (*SADABS*; Krause *et al.*, 2015 ▸)
*T*_min_, *T*_max_	0.815, 1.000
No. of measured, independent and observed [*I* > 2σ(*I*)] reflections	209395, 3747, 3658
*R*_int_	0.039
(sin θ/λ)_max_ (Å^−1^)	0.667

Refinement	
*R*[*F*^2^ > 2σ(*F*^2^)], *wR*(*F*^2^), *S*	0.015, 0.035, 1.08
No. of reflections	3747
No. of parameters	181
H-atom treatment	H-atom parameters constrained
Δρ_max_, Δρ_min_ (e Å^−3^)	0.40, −0.41

Computer programs: *APEX5* (Bruker, 2023 ▸), *SAINT-Plus* (Bruker, 2012 ▸), *SHELXS2014/5* (Sheldrick, 2015*a* ▸), *SHELXL2014/5* (Sheldrick, 2015*b* ▸), *ShelXle* (Hübschle *et al.*, 2011 ▸), *ORTEP-3 for Windows* (Farrugia, 2012 ▸), *Mercury* (Macrae *et al.*, 2020 ▸), *CrystalExplorer* (Spackman *et al.*, 2021 ▸), *OLEX2* (Dolomanov *et al.*, 2009 ▸), *PLATON* (Spek, 2020 ▸), and *publCIF* (Westrip, 2010 ▸).

## supplementary crystallographic information

### {2-[Bis(pyridin-2-ylmethyl)amino]ethane-1-thiolato}chloridocadmium(II) Crystal data

**Table:**

[Cd(C_14_H_16_N_3_S)Cl]	*F*(000) = 808
*M**_r_* = 406.21	*D*_x_ = 1.783 Mg m^−^^3^
Monoclinic, *P*2_1_/*c*	Mo *K*α radiation, λ = 0.71073 Å
*a* = 12.8451 (14) Å	Cell parameters from 9498 reflections
*b* = 7.4508 (7) Å	θ = 2.6–28.3°
*c* = 15.9284 (17) Å	µ = 1.75 mm^−^^1^
β = 96.965 (3)°	*T* = 100 K
*V* = 1513.2 (3) Å^3^	Block, colourless
*Z* = 4	0.37 × 0.31 × 0.24 mm

### {2-[Bis(pyridin-2-ylmethyl)amino]ethane-1-thiolato}chloridocadmium(II) Data collection

**Table:**

Bruker D8 Venture Photon 3 diffractometer	3747 independent reflections
Radiation source: Imus	3658 reflections with *I* > 2σ(*I*)
Multi-layer optics monochromator	*R*_int_ = 0.039
ω and ψ scans	θ_max_ = 28.3°, θ_min_ = 2.6°
Absorption correction: multi-scan (*SADABS*; Krause *et al.*, 2015)	*h* = −17→17
*T*_min_ = 0.815, *T*_max_ = 1.000	*k* = −9→9
209395 measured reflections	*l* = −21→21

### {2-[Bis(pyridin-2-ylmethyl)amino]ethane-1-thiolato}chloridocadmium(II) Refinement

**Table:**

Refinement on *F*^2^	Primary atom site location: other
Least-squares matrix: full	Hydrogen site location: inferred from neighbouring sites
*R*[*F*^2^ > 2σ(*F*^2^)] = 0.015	H-atom parameters constrained
*wR*(*F*^2^) = 0.035	*w* = 1/[σ^2^(*F*_o_^2^) + (0.0072*P*)^2^ + 1.2877*P*] where *P* = (*F*_o_^2^ + 2*F*_c_^2^)/3
*S* = 1.08	(Δ/σ)_max_ = 0.003
3747 reflections	Δρ_max_ = 0.40 e Å^−^^3^
181 parameters	Δρ_min_ = −0.41 e Å^−^^3^
0 restraints	

### {2-[Bis(pyridin-2-ylmethyl)amino]ethane-1-thiolato}chloridocadmium(II) Special details

**Table:**

Geometry. All esds (except the esd in the dihedral angle between two l.s. planes) are estimated using the full covariance matrix. The cell esds are taken into account individually in the estimation of esds in distances, angles and torsion angles; correlations between esds in cell parameters are only used when they are defined by crystal symmetry. An approximate (isotropic) treatment of cell esds is used for estimating esds involving l.s. planes.

### {2-[Bis(pyridin-2-ylmethyl)amino]ethane-1-thiolato}chloridocadmium(II) Fractional atomic coordinates and isotropic or equivalent isotropic displacement parameters (Å^2^)

**Table:**

	*x*	*y*	*z*	*U*_iso_*/*U*_eq_	
Cd1	0.74709 (2)	0.31187 (2)	0.58883 (2)	0.01483 (3)	
Cl1	0.70287 (3)	0.08478 (4)	0.47746 (2)	0.02385 (7)	
S1	0.84107 (3)	0.20129 (4)	0.72345 (2)	0.02084 (7)	
N1	0.75863 (8)	0.60172 (14)	0.66540 (6)	0.0154 (2)	
N1A	0.57698 (8)	0.41020 (15)	0.59984 (7)	0.0183 (2)	
N1B	0.83627 (8)	0.51728 (14)	0.51512 (6)	0.01542 (19)	
C1A	0.49427 (11)	0.29875 (19)	0.58949 (9)	0.0208 (3)	
H1A	0.501646	0.186315	0.562702	0.025*	
C1B	0.86110 (10)	0.48024 (17)	0.43735 (8)	0.0172 (2)	
H1B	0.838155	0.369821	0.411608	0.021*	
C1C	0.85864 (10)	0.41625 (18)	0.77926 (8)	0.0199 (2)	
H1CA	0.863686	0.393122	0.840820	0.024*	
H1CB	0.926067	0.469376	0.767630	0.024*	
C2A	0.39857 (11)	0.3412 (2)	0.61632 (9)	0.0236 (3)	
H2A	0.341147	0.260232	0.607530	0.028*	
C2B	0.91853 (10)	0.59597 (18)	0.39343 (8)	0.0185 (2)	
H2B	0.936190	0.565033	0.339052	0.022*	
C2C	0.77164 (10)	0.55322 (18)	0.75572 (8)	0.0180 (2)	
H2CA	0.704677	0.503679	0.770438	0.022*	
H2CB	0.787401	0.663183	0.789789	0.022*	
C3A	0.38858 (10)	0.5042 (2)	0.65621 (9)	0.0254 (3)	
H3A	0.324587	0.535668	0.676714	0.030*	
C3B	0.94985 (10)	0.75858 (19)	0.43059 (8)	0.0211 (2)	
H3B	0.989827	0.841014	0.402160	0.025*	
C4A	0.47328 (10)	0.6211 (2)	0.66585 (9)	0.0225 (3)	
H4A	0.467619	0.734369	0.692343	0.027*	
C4B	0.92207 (10)	0.79909 (17)	0.50963 (8)	0.0193 (2)	
H4B	0.940726	0.911505	0.535305	0.023*	
C5A	0.56633 (10)	0.57087 (18)	0.63637 (8)	0.0179 (2)	
C5B	0.86663 (9)	0.67381 (16)	0.55110 (8)	0.0148 (2)	
C6A	0.65923 (10)	0.69587 (17)	0.64093 (8)	0.0188 (2)	
H6AA	0.661166	0.753150	0.585060	0.023*	
H6AB	0.650871	0.791863	0.682566	0.023*	
C6B	0.84680 (10)	0.70666 (17)	0.64152 (8)	0.0174 (2)	
H6BA	0.910811	0.675772	0.679852	0.021*	
H6BB	0.832371	0.835846	0.648930	0.021*	

### {2-[Bis(pyridin-2-ylmethyl)amino]ethane-1-thiolato}chloridocadmium(II) Atomic displacement parameters (Å^2^)

**Table:**

	*U*^11^	*U*^22^	*U*^33^	*U*^12^	*U*^13^	*U*^23^
Cd1	0.01714 (5)	0.01179 (5)	0.01600 (5)	−0.00086 (3)	0.00376 (3)	−0.00072 (3)
Cl1	0.03070 (16)	0.01655 (14)	0.02427 (15)	−0.00161 (12)	0.00322 (12)	−0.00640 (11)
S1	0.02707 (16)	0.01780 (15)	0.01783 (14)	0.00230 (12)	0.00344 (12)	0.00358 (11)
N1	0.0162 (5)	0.0151 (5)	0.0152 (5)	−0.0013 (4)	0.0032 (4)	−0.0003 (4)
N1A	0.0181 (5)	0.0178 (5)	0.0191 (5)	−0.0005 (4)	0.0028 (4)	−0.0011 (4)
N1B	0.0163 (5)	0.0145 (5)	0.0154 (5)	−0.0001 (4)	0.0015 (4)	0.0000 (4)
C1A	0.0200 (6)	0.0215 (6)	0.0209 (6)	−0.0024 (5)	0.0019 (5)	−0.0014 (5)
C1B	0.0176 (5)	0.0172 (6)	0.0163 (5)	0.0012 (4)	0.0001 (4)	−0.0018 (4)
C1C	0.0220 (6)	0.0219 (6)	0.0157 (5)	−0.0015 (5)	0.0019 (4)	0.0012 (5)
C2A	0.0172 (6)	0.0301 (7)	0.0231 (6)	−0.0033 (5)	0.0007 (5)	0.0014 (5)
C2B	0.0176 (5)	0.0229 (6)	0.0153 (5)	0.0027 (5)	0.0026 (4)	0.0005 (5)
C2C	0.0201 (6)	0.0206 (6)	0.0140 (5)	−0.0028 (5)	0.0044 (4)	−0.0020 (5)
C3A	0.0158 (6)	0.0361 (8)	0.0243 (6)	0.0033 (5)	0.0023 (5)	0.0002 (6)
C3B	0.0215 (6)	0.0218 (6)	0.0206 (6)	−0.0025 (5)	0.0050 (5)	0.0044 (5)
C4A	0.0199 (6)	0.0246 (7)	0.0226 (6)	0.0056 (5)	0.0015 (5)	−0.0031 (5)
C4B	0.0220 (6)	0.0157 (6)	0.0206 (6)	−0.0027 (5)	0.0035 (5)	0.0001 (5)
C5A	0.0178 (6)	0.0189 (6)	0.0168 (5)	0.0016 (5)	0.0011 (4)	0.0000 (5)
C5B	0.0141 (5)	0.0145 (5)	0.0158 (5)	0.0011 (4)	0.0015 (4)	0.0004 (4)
C6A	0.0196 (6)	0.0148 (6)	0.0222 (6)	0.0016 (4)	0.0031 (5)	−0.0013 (5)
C6B	0.0210 (6)	0.0156 (6)	0.0163 (5)	−0.0051 (5)	0.0045 (4)	−0.0022 (4)

### {2-[Bis(pyridin-2-ylmethyl)amino]ethane-1-thiolato}chloridocadmium(II) Geometric parameters (Å, º)

**Table:**

Cd1—N1A	2.3313 (11)	C2A—C3A	1.384 (2)
Cd1—N1B	2.3151 (11)	C2A—H2A	0.9500
Cd1—N1	2.4758 (11)	C2B—C3B	1.3865 (19)
Cd1—S1	2.4710 (4)	C2B—H2B	0.9500
Cd1—Cl1	2.4674 (4)	C2C—H2CA	0.9900
S1—C1C	1.8324 (14)	C2C—H2CB	0.9900
N1—C6B	1.4639 (15)	C3A—C4A	1.388 (2)
N1—C6A	1.4676 (16)	C3A—H3A	0.9500
N1—C2C	1.4730 (15)	C3B—C4B	1.3828 (18)
N1A—C1A	1.3427 (17)	C3B—H3B	0.9500
N1A—C5A	1.3452 (17)	C4A—C5A	1.3876 (18)
N1B—C5B	1.3366 (16)	C4A—H4A	0.9500
N1B—C1B	1.3447 (16)	C4B—C5B	1.3889 (17)
C1A—C2A	1.3862 (19)	C4B—H4B	0.9500
C1A—H1A	0.9500	C5A—C6A	1.5085 (18)
C1B—C2B	1.3792 (18)	C5B—C6B	1.5126 (17)
C1B—H1B	0.9500	C6A—H6AA	0.9900
C1C—C2C	1.5261 (18)	C6A—H6AB	0.9900
C1C—H1CA	0.9900	C6B—H6BA	0.9900
C1C—H1CB	0.9900	C6B—H6BB	0.9900
			
N1A—Cd1—N1B	110.87 (4)	C1B—C2B—H2B	120.8
N1A—Cd1—N1	71.91 (4)	C3B—C2B—H2B	120.8
N1A—Cd1—S1	113.78 (3)	N1—C2C—C1C	113.39 (10)
N1A—Cd1—Cl1	97.67 (3)	N1—C2C—H2CA	108.9
N1B—Cd1—N1	70.70 (4)	C1C—C2C—H2CA	108.9
N1B—Cd1—S1	116.16 (3)	N1—C2C—H2CB	108.9
N1B—Cd1—Cl1	100.00 (3)	C1C—C2C—H2CB	108.9
S1—Cd1—N1	82.51 (3)	H2CA—C2C—H2CB	107.7
Cl1—Cd1—N1	161.29 (3)	C2A—C3A—C4A	119.13 (13)
Cl1—Cd1—S1	116.159 (13)	C2A—C3A—H3A	120.4
C1C—S1—Cd1	98.51 (4)	C4A—C3A—H3A	120.4
C6B—N1—C6A	110.56 (10)	C4B—C3B—C2B	119.07 (12)
C6B—N1—C2C	112.65 (10)	C4B—C3B—H3B	120.5
C6A—N1—C2C	111.64 (10)	C2B—C3B—H3B	120.5
C6B—N1—Cd1	109.87 (7)	C5A—C4A—C3A	119.27 (13)
C6A—N1—Cd1	106.73 (7)	C5A—C4A—H4A	120.4
C2C—N1—Cd1	105.07 (7)	C3A—C4A—H4A	120.4
C1A—N1A—C5A	118.89 (11)	C3B—C4B—C5B	119.35 (12)
C1A—N1A—Cd1	122.09 (9)	C3B—C4B—H4B	120.3
C5A—N1A—Cd1	117.23 (8)	C5B—C4B—H4B	120.3
C5B—N1B—C1B	118.99 (11)	N1A—C5A—C4A	121.57 (12)
C5B—N1B—Cd1	119.73 (8)	N1A—C5A—C6A	116.68 (11)
C1B—N1B—Cd1	121.25 (8)	C4A—C5A—C6A	121.72 (12)
N1A—C1A—C2A	122.62 (13)	N1B—C5B—C4B	121.48 (11)
N1A—C1A—H1A	118.7	N1B—C5B—C6B	118.39 (11)
C2A—C1A—H1A	118.7	C4B—C5B—C6B	119.96 (11)
N1B—C1B—C2B	122.69 (12)	N1—C6A—C5A	112.04 (10)
N1B—C1B—H1B	118.7	N1—C6A—H6AA	109.2
C2B—C1B—H1B	118.7	C5A—C6A—H6AA	109.2
C2C—C1C—S1	114.95 (9)	N1—C6A—H6AB	109.2
C2C—C1C—H1CA	108.5	C5A—C6A—H6AB	109.2
S1—C1C—H1CA	108.5	H6AA—C6A—H6AB	107.9
C2C—C1C—H1CB	108.5	N1—C6B—C5B	112.70 (10)
S1—C1C—H1CB	108.5	N1—C6B—H6BA	109.1
H1CA—C1C—H1CB	107.5	C5B—C6B—H6BA	109.1
C3A—C2A—C1A	118.48 (13)	N1—C6B—H6BB	109.1
C3A—C2A—H2A	120.8	C5B—C6B—H6BB	109.1
C1A—C2A—H2A	120.8	H6BA—C6B—H6BB	107.8
C1B—C2B—C3B	118.37 (12)		
			
C5A—N1A—C1A—C2A	−1.2 (2)	C3A—C4A—C5A—N1A	−1.1 (2)
Cd1—N1A—C1A—C2A	163.14 (10)	C3A—C4A—C5A—C6A	176.77 (12)
C5B—N1B—C1B—C2B	1.21 (18)	C1B—N1B—C5B—C4B	0.69 (18)
Cd1—N1B—C1B—C2B	−176.79 (9)	Cd1—N1B—C5B—C4B	178.72 (9)
Cd1—S1—C1C—C2C	31.48 (9)	C1B—N1B—C5B—C6B	−174.55 (11)
N1A—C1A—C2A—C3A	−0.8 (2)	Cd1—N1B—C5B—C6B	3.49 (14)
N1B—C1B—C2B—C3B	−1.37 (19)	C3B—C4B—C5B—N1B	−2.37 (19)
C6B—N1—C2C—C1C	−69.38 (14)	C3B—C4B—C5B—C6B	172.79 (12)
C6A—N1—C2C—C1C	165.53 (10)	C6B—N1—C6A—C5A	160.44 (10)
Cd1—N1—C2C—C1C	50.21 (11)	C2C—N1—C6A—C5A	−73.31 (13)
S1—C1C—C2C—N1	−59.72 (13)	Cd1—N1—C6A—C5A	40.98 (11)
C1A—C2A—C3A—C4A	1.8 (2)	N1A—C5A—C6A—N1	−42.59 (15)
C1B—C2B—C3B—C4B	−0.36 (19)	C4A—C5A—C6A—N1	139.47 (12)
C2A—C3A—C4A—C5A	−0.9 (2)	C6A—N1—C6B—C5B	−85.38 (13)
C2B—C3B—C4B—C5B	2.2 (2)	C2C—N1—C6B—C5B	148.94 (11)
C1A—N1A—C5A—C4A	2.09 (19)	Cd1—N1—C6B—C5B	32.17 (12)
Cd1—N1A—C5A—C4A	−162.98 (10)	N1B—C5B—C6B—N1	−25.47 (16)
C1A—N1A—C5A—C6A	−175.86 (12)	C4B—C5B—C6B—N1	159.22 (11)
Cd1—N1A—C5A—C6A	19.08 (14)		

### {2-[Bis(pyridin-2-ylmethyl)amino]ethane-1-thiolato}chloridocadmium(II) Hydrogen-bond geometry (Å, º)

**Table:**

*D*—H···*A*	*D*—H	H···*A*	*D*···*A*	*D*—H···*A*
C2*A*—H2*A*···Cl1^i^	0.95	2.93	3.6784 (15)	137
C2*B*—H2*B*···S1^ii^	0.95	2.88	3.5459 (14)	128
C6*B*—H6*BB*···S1^iii^	0.99	2.97	3.9133 (13)	160

Symmetry codes: (i) −*x*+1, −*y*, −*z*+1; (ii) *x*, −*y*+1/2, *z*−1/2; (iii) *x*, *y*+1, *z*.
